# The Effect of Molar Mass and Charge Density on the Formation of Complexes between Oppositely Charged Polyelectrolytes

**DOI:** 10.3390/polym9020050

**Published:** 2017-02-04

**Authors:** Feriel Meriem Lounis, Joseph Chamieh, Laurent Leclercq, Philippe Gonzalez, Hervé Cottet

**Affiliations:** Institut des Biomolécules Max Mousseron, IBMM, UMR 5247 CNRS, Université de Montpellier, Ecole Nationale Supérieure de Chimie de Montpellier, Place Eugène Bataillon, CC 1706, 34095 Montpellier CEDEX 5, France; feriel-meriem.lounis@etu.umontpellier.fr (F.M.L.); Joseph.chamieh@umontpellier.fr (J.C.); Laurent.leclercq@umontpellier.fr (L.L.); Philippe.gonzalez@univ-montp2.fr (P.G.)

**Keywords:** polyelectrolyte complexes, molar mass, frontal analysis continuous capillary electrophoresis, counter-ion release, binding constants, ionic strength dependence

## Abstract

The interactions between model polyanions and polycations have been studied using frontal continuous capillary electrophoresis (FACCE) which allows the determination of binding stoichiometry and binding constant of the formed polyelectrolyte complex (PEC). In this work, the effect of the poly(l-lysine) (PLL) molar mass on the interaction with statistical copolymers of acrylamide and 2-acrylamido-2-methyl-1-propanesulfonate (PAMAMPS) has been systematically investigated for different PAMAMPS chemical charge densities (15% and 100%) and different ionic strengths. The study of the ionic strength dependence of the binding constant allowed the determination of the total number of released counter-ions during the formation of the PEC, which can be compared to the total number of counter-ions initially condensed on the individual polyelectrolyte partners before the association. Interestingly, this fraction of released counter-ions, which was strongly dependent on the PLL molar mass, was almost independent of the PAMAMPS charge density. These findings are useful to predict the binding constant according to the molar mass and charge density of the polyelectrolyte partners.

## 1. Introduction

Within the last decades, the formation and properties of polyelectrolyte complexes (PEC) have been widely investigated from an experimental and a theoretical point of view [1,2,3,4,5,6,7,8] leading to the development of interesting industrial [9,10,11], biological [12,13] or biotechnological [14,15,16,17,18] applications of PEC. These investigations revealed that a considerable number of factors related to the polyelectrolytes structural features (charge density, distribution of the charges on polyelectrolyte backbone, molar masses), preparation of polyelectrolyte mixtures (titration or solution jet) and environmental parameters (ionic strength, pH, and temperature) influence the formation of PEC. 

Depending on the polyelectrolyte nature, different PEC structures were obtained when the molar masses were varied (fluid or gelled systems [19], soluble PEC [20], insoluble and highly aggregated systems [21]). Furthermore, the molar mass had a major impact on the particle size of the PEC [22,23,24] and the thermodynamic binding parameters [25,26,27,28]. Priftis et al. [26] used two different molar masses (degrees of polymerization of 100 and 400) of poly(l-ornithine hydrobromide) and poly(l-glutamic acid) (PO-PGlu) mixtures to examine their effect on the thermodynamic characteristics as reflected by isothermal titration calorimetry (ITC). Considering the complex coacervation phenomenon, an increase of the molar mass of the used polyelectrolytes was expected to result in a denser phase and a more extended coacervate domains in phase diagram. The enthalpic and entropic changes corresponding to ion pairing step were higher when polypeptides with higher molar mass were used [26]. Using ITC, Maurstad et al. [27] showed that the interaction between xanthan and chitosan depends on the chain length of both polyelectrolytes. Increasing the chain length of chitosan led to more complicated ITC curves, consisting of two different stages with both stages being endothermic. Fitting the stages with the one-site model [29,30] separately gave a low association constant indicative of non-specific interaction. However, no strong differences have been reported when varying the molar masses of interacting carboxymethyl pullulan and dextran [31] or between poly(diallyldimethyl ammonium chloride) (PDADMAC) and poly(sodium acrylate) (PANa) [32]. Only slight differences on binding parameters according to the nature of the injectant were confirmed [31,32].

In a previous paper [33], we presented the experimental data about the effect of the chemical charge density of statistical copolymers of acrylamide and 2-acrylamido-2-methyl-l-propanesulfonate (PAMAMPS) interacting with poly(l-lysine) (PLL, degree of polymerization of 50), at different ionic strengths. In this work, we present the effect of PLL molar mass (*DP^+^* of 20, 50, 100, 250) on the interaction with PAMAMPS having different chemical charge densities of 15% and 100%. This study represents a quantitative investigation about how the ionic strength, the molar mass of the polycation and the chemical charge density of the polyanion influence the thermodynamic binding parameters when PEC are formed. Binding site constant and binding stoichiometry were systematically measured by frontal analysis continuous capillary electrophoresis (FACCE), which is a performant technique for the determination of binding parameters between polyelectrolytes and biomolecules [34], proteins [35,36,37,38], and bacteria [39]. The experimental data obtained by FACCE were interpreted using the theoretical model of independent identical interacting sites and via the experimental determination of the number of released counter-ions after the PEC formation.

## 2. Theoretical Section

In this study, PAMAMPS chains are considered as the substrate molecules (*S*) carrying *n* identical interacting sites *–s* having the same energy. Each interacting site –*s* is defined as a fragment of PAMAMPS chains. A PAMAMPS polyanionic chain has a degree of polymerization *DP*^−^ and a chemical charge densities *f*, defined as the molar ratio in charged monomers (AMPS) to total monomers. PLL chains with a degree of polymerization *DP^+^* corresponding to the number of charges (number of lysine residues) are considered as the ligand molecules (*L*). 

Each binding site *–s* may interact with one PLL chain yielding to one bound site according to equilibrium (1):

(1) $$-s+\mathrm{PLL}\overset{k}{⇄}\mathrm{PLL}-s$$

*k* is the binding site constant defined according to the mass action law as:

(2) $$k=\frac{\left[PLL-s\right]}{[-s]\left[PLL\right]}$$

[*PLL-s*], [*–s*], and [*PLL*] are the concentrations of bound sites, free sites and free PLL chains. The average number of bound ligands (PLL) per introduced substrate (PAMAMPS) $\bar{n}$ can be calculated from Equation (3):

(3) $$\bar{n}=\frac{{\left[PLL\right]}_{0}-[PLL]}{{\left[PAMAMPS\right]}_{0}}$$

where [*PLL*]_0_ and [*PAMAMPS*]_0_ are the initial concentrations in PLL and PAMAMPS chains, respectively. The average number of bound ligands by introduced substrate is related to the binding parameters *k* and *n* in the case of the model of identical sites by the following equation:

(4) $$\bar{n}=\frac{nk[PLL]}{1+k[PLL]}$$

The free ligand (free PLL) concentration can be experimentally determined by FACCE (as described in experimental section) for different initial molar ratios [*PLL*]_0_/[*PAMAMPS*]_0_. The isotherm of adsorption is defined as the plot of $\bar{n}$ versus [*PLL*]. This experimental isotherm can be directly fitted using Equation (4) by adjusting *n* and *k* values. The equilibrium associated to the full binding of the *n* sites of the PAMAMPS is given by:

(5) $$\mathrm{PAMAMPS}+n\mathrm{PLL}\overset{\beta_{n}}{⇄}{\text{PAMAMPS-PLL}}_{n}$$

β*_n_* is the global constant of equilibrium (5) and is related to the binding site constant using Equation (6):

(6) $$\beta_{n}=k^{n}$$

According to the Manning condensation theory [40,41], the number of the counter-ions Na^+^ initially condensed onto a PAMAMPS chain *N*_Na_^+^ (respectively, the number of the counter-ions Cl^−^ initially condensed on a PLL chain *N*_Cl_^−^) are given by Equations (7) and (8):

(7) $$N_{{\mathrm{Na}}^{+}}=\theta^{-}\times {\mathrm{DP}}^{-}\times f$$

(8) $$N_{{\mathrm{Cl}}^{-}}=\theta^{+}\times {\mathrm{DP}}^{+}$$

where θ*^−^* and θ*^+^* are the fractions of condensed charged monomers on PAMAMPS and PLL chains, respectively. As predicted by the Manning theory and verified experimentally by Ibrahim et al. [42], θ*^+^* = 0.5 (resp. 0.35) for PLL chains with *DP^+^* = 250, 100 and 50 (resp. 20). As for PAMAMPS chains with a chemical charge density *f* ≤ 35%, θ*^−^* = 0, and for PAMAMPS chains with a chemical charge density *f* > 35%, θ*^−^* is calculated by $\theta^{-}=1-\frac{0.35}{f}$ [43]. The total number of condensed counter-ions *N*_counter-ion_ constituting the entropic reservoir before the PEC formation, corresponds to the number of counter-ions associated with *n* PLL chains and one PAMAMPS chain, according to:

(9) $$N_{\mathrm{conter}\;\mathrm{ions}}=N_{{\mathrm{Na}}^{+}}+n\times N_{{\mathrm{Cl}}^{-}}=\theta^{-}\times DP^{-}\times f+n\times \theta^{+}\times DP^{+}$$

It is worth noting that the estimation of *N*_counter-ions_ within the framework of the Manning condensation theory presents some limitations. Indeed, Trizac et al. [44] numerically demonstrated that, at finite ionic strength, the critical charge density parameter ξ^*^ (above which condensation occurs) decreases with increasing ionic strength. For instance, at 1M ionic strength for PAMAMPS, we can evaluate from the work of Trizac et al. [44] a shift of ξ^*^ from unity (Manning) to ~0.65. Moreover, Carrillo et al. [45] showed using hybrid Monte-Carlo/molecular dynamics simulations that the fraction of condensed counter-ions decreases with increasing ionic strength, while Muthukumar [46] predicted an opposite behavior. Due to the complexity of the counter-ions condensation phenomenon, which depends on many physico-chemical parameters (ionic strength, polymer concentration, polymer backbone size, charge density parameter), we restricted our estimation to the framework of Manning theory.

This total number of counter-ions can be compared to the effective number of released counter-ions during the PEC formation, which can be experimentally determined from the slope of log β*_n_* vs. log *I* dependence (mathematically expressed as $-\frac{\partial \;n\log k}{\partial \left[{\mathrm{Na}}^{+},{\mathrm{Cl}}^{-}\right]}$), where *I* is the ionic strength of the background electrolyte which is controlled by the NaCl concentration [*Na^+^, Cl^−^*] [47,48,49,50,51].

## 3. Materials and Methods

### 3.1. Chemicals

Random copolymers of acrylamide and 2-acrylamido-2-methyl-1-propanesulfonate (PAMAMPS) with chemical charge densities of 15% and 100% were synthesized by free radical copolymerization. The physico-chemical properties of these copolymers (molar masses, polydispersity indexes and chemical charge density distributions) were determined as described in a previous work [52]. Poly-l-Lysine (PLL) with different degrees of polymerization (*DP*^+^ = 20, 50, 100, 250 corresponding to *M*_w_ = 3300, 8200, 16,000, 41,000 g/mol molar masses respectively and polydispersity indexes between 1.0 and 1.2) were supplied by Alamanda Polymers (Huntsville, AL, USA). Poly(diallyldimethyl ammonium chloride) (PDADMAC), *M*_w_ = 400–500 kDa, was purchased from Sigma Aldrich (St Quentin Fallavier, France). Tris hydroxymethylaminomethane (CH_2_OH)_3_CNH_2_, 99.9% was purchased from Merck (Darmstadt, Germany). Hydrochloric acid 37%, sodium hydroxide and sodium chloride were purchased from VWR (Leuven, Belgium). Deionized water was further purified using a Milli-Q system (Millipore, Molsheim, France). All chemical were used without any further purification.

### 3.2. Preparation of the PEC Mixtures

PAMAMPS and PLL stock solutions were prepared in Tris-HCl buffer pH 7.4 (12 mM Tris, 10 mM HCl and appropriate amount of NaCl) at room temperature. The ionic strength of the buffer was adjusted by adding adequate amounts of NaCl. The concentrations of stock solutions were 1.14, 3.2, 5 g/L for PAMAMPS 100%, PAMAMPS 15% and PLL respectively. PLL diluted solution, with concentrations from 0.1 to 4 g/L for PLL-PAMAMPS mixtures and from 0.1 to 2 g/L for the calibration curve, were prepared by diluting in the same Tris-HCl-NaCl buffer. PLL-PAMAMS mixtures were prepared by adding 100 µL of PAMAMPS stock solutions to 100 µL of PLL solutions (See Tables S1 and S2 in the supporting information for the concentrations of PAMAMPS and PLL in the mixtures). The final mixtures of a volume of 200 µL were equilibrated by homogenizing with a vortex stirrer during 1 min and analyzed by FACCE as described below.

### 3.3. FACCE Procedure

FACCE experiments were carried out using a 3D-Agilent Technologies system (Waldbronn, Germany) equipped with a diode array detector set at 200 nm (band width: 16 nm). Bare fused silica capillaries (50 µm i.d. × 33.5 cm (8.5 cm to the detector)) were purchased from Polymicro Technologies (Photonlines, Saint-Germain-en-Laye, France). New capillaries were first flushed with a 1.0 M NaOH solution for 30 min, and then with purified water for 20 min. A PDADMAC solution of 0.2% *w*/*w* was prepared in 2 M NaCl solution and used for capillary coating by flushing the new capillaries for 20 min. The capillaries were pre-conditioned before each run according to the following procedure: water for 2 min, PDADMAC 0.2% *w*/*w* in water for 3 min, Tris-HCl-NaCl buffer for 3 min. The temperature of the capillary was maintained at 25 °C. Free PLL molecules in the equilibrated mixtures were continuously and electrokinetically introduced in the capillary by applying a continuous positive polarity voltage of 1 kV and a co-pressure of 5 mbar. These applied voltage and co-pressure allowed the entrance and the quantification of the free PLL (*L*) while avoiding the entrance of PAMAMPS and PEC. All samples were placed at the capillary end which is the closest from the detection point (8.5 cm) to reduce the migration times.

## 4. Results

**Figure 1:**
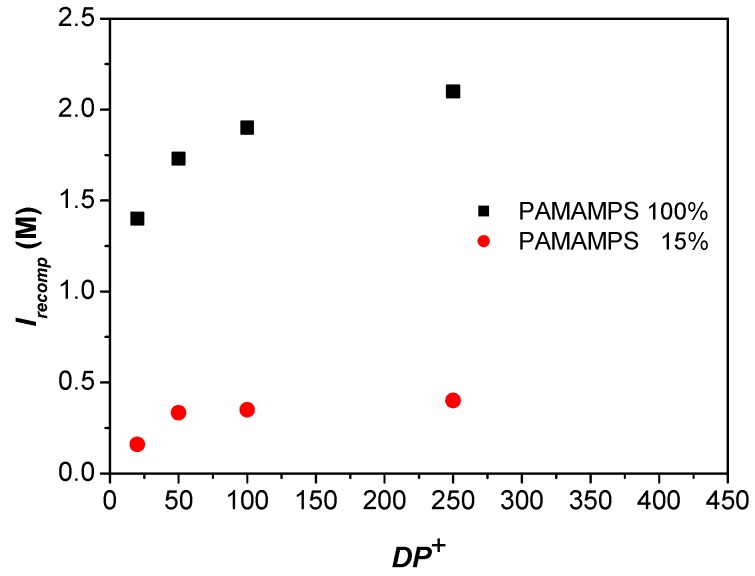
Variation of the ionic strength of recomplexation *I*_recomp_ as a function the degree of polymerization of PLL (*DP^+^* = 20, 50, 100, 250) for two PAMAMPS charge densities (15% and 100%).

To investigate the effect of PLL molar mass on the PEC formation, isotherms of adsorption were plotted for different molar masses of PLL (*DP*^+^ 20, 50, 100, 250) in interaction with PAMAMPS of two different charge densities (*f* = 100% and 15%). For each PLL molar mass and each PAMAMPS charge density, at least three different ionic strengths were investigated (in total 29 isotherms of adsorption were plotted) to get the ionic strength dependence of the binding constant. However, for the success of the experimental determination of binding parameters [33,47], it was important to adjust the range of ionic strength according to the PLL molar mass and PAMAMPS charge density (0.85–1.728 M for *f* = 100%; 0.15–0.39 M for *f* = 15%; see Table 1 and Table 2 for the investigated ionic strength ranges). Measurable binding site constants were obtained when the ionic strength ranged from 45% to 90% of the ionic strength of recomplexation *I*_recomp_ [52]. At lower ionic strengths, the binding constants were too high to be measured by FACCE, while at higher ionic strengths (close to *I*_recomp_), the probability of dissociation of the complex was increased. The ionic strength of recomplexation *I*_recomp_ is defined as the salt concentration at which a solid PLL-PAMAMPS complex previously destabilized at high ionic strength, re-formed when water was added. *I*_recomp_ was determined by turbidimetry as described in a previous work [52]. As expected, and as displayed in Figure 1, *I*_recomp_ was found to increase with the polycation molar mass *M*_w_, in good agreement with the literature [53]. The increase of *I*_recomp_ with the PLL molar mass is much higher for PAMAMPS 100% than for 15%. *I*_recomp_ are, of course, higher for PAMAMPS 100% than for PAMAMPS 15%, which means that the PEC are much stronger with PAMAMPS 100% than with PAMAMPS 15%. 

**Figure 2:**
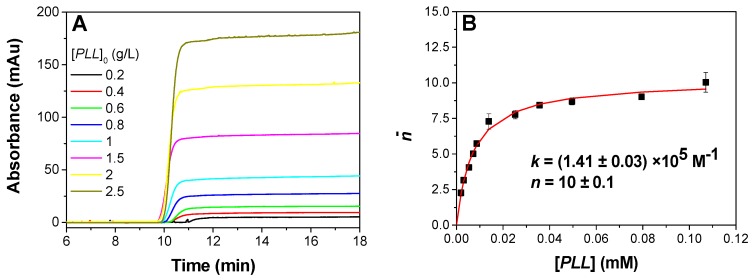
Example of determination of interaction stoichiometry (*n*) and binding site constant (*k*) by FACCE for the interaction PLL100-PAMAMPS 15% at 315 mM ionic strength. Electropherograms obtained by FACCE for the PLL100-PAMAMPS 15% equilibrated mixtures (**A**); and the corresponding isotherm of adsorption giving the number of bound PLL100 chains per PAMAMPS 15% chain as a function of the free PLL100 concentration (**B**). Experimental conditions: PDADMAC coated capillary 33.5 cm (8.5 cm to the detector) × 50 µm i.d. Background electrolyte: 12 mM Tris, 10 mM HCl, 305 mM NaCl, pH 7.4. Applied voltage: +1 kV with a co-hydrodynamic pressure of +5 mbar. Detection at 200 nm. Samples were prepared in the background electrolyte by 50/50 *v*/*v* dilution of the following solutions: PAMAMPS 15% at 3.2 g/L with PLL100 at 5, 4, 3, 2.5, 2, 1.6, 1.2, 1, 0.8, 0.6, 0.4 g/L.

Binding parameters where obtained by plotting the isotherm of adsorption using FACCE as previously described [33,35,36]. In brief, for each (PLL/PAMAMPS/ionic strength) triplet, the free PLL concentrations in the equilibrated mixtures containing different PLL/PAMAMPS ratios were determined by FACCE. Examples of electropherograms obtained by FACCE for PLL100 in presence of PAMAMPS 15% at *I* = 315 mM are given in Figure 2A, where front heights are proportional to the free PLL100 concentration [*PLL*] at equilibrium in the mixture. Isotherms of adsorption were plotted using Equation (3) by representing the number of bound PLL ligands per PAMAMPS substrate molecules $\bar{n}$ versus the free PLL ligand. The isotherms of adsorption were fitted by non-linear least-square routine on Microsoft Excel, using the model of identical interacting sites presented in Equation (4) yielding the determination of the binding site constant *k*, and the stoichiometry of interaction *n* (expressed in term of PLL chains bound per total PAMAMPS). Figure 2B shows an example of isotherm of adsorption and the non-linear fitting corresponding to data presented in Figure 2A. All the other isotherms of adsorption and the corresponding curve fitting are provided in the supporting information (see Figures S1–S8).

### 4.1. Influence of the Ionic Strength on PEC Stoichiometry

**Figure 3:**
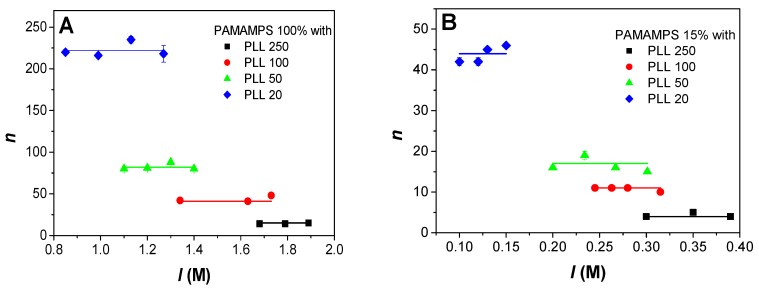
Variation of the interaction stoichiometry *n*, expressed as the number of PLL chains per PAMAMPS chain, as a function of the ionic strength *I* for the interactions between PLL of different degrees of polymerization (20, 50, 100, 250) and PAMAMPS 100% (**A**), or PAMAMPS 15% (**B**).

The stoichiometry of interactions, *n,* corresponding to the maximum number of PLL chains bound per PAMAMPS chains at saturation of the isotherm is displayed in Figure 3A for PAMAMPS 100%, and in Figure 3B for PAMAMPS 15%. It can be observed that the stoichiometry *n* was independent of the ionic strength, whatever the degree of polymerization of the PLL and the chemical charge density of the PAMAMPS. As expected, the stoichiometry decreased when the degree of polymerization of PLL increased, since a lower number of PLL chain is required to “neutralize” the PAMAMPS substrate. On average, expressed in terms of a number of Lys residues per PAMAMPS chain, the stoichiometry is similar whatever the *DP^+^* of the PLL and is about 4000 for PAMAMPS 100% and about 800 for PAMAMPS 15%. This number of Lys residues at saturation on the PAMAMPS substrate is obviously much higher for the PAMAMPS 100%, but not in the ratio of the charge densities (4000/800 = 5 is lower than 100%/15% = 6.7). This difference is due to the difference in charge composition of the PEC according to the PAMAMPS charge density. The charge stoichiometry (*n*_(Lys/AMPS)_) is given in Table 1 and Table 2 for the PAMAMPS 100% and 15%, respectively. *n*_(Lys/AMPS)_ tends to ~1 for PAMAMPS 100% whatever the PLL *DP^+^*; while it tends to ~2 for PAMAMPS 15%. These results are in good agreement with the general rule established in previous investigations concerning the measurement of charge stoichiometries by ^1^H-NMR [52]. This rules states that the PEC stoichiometry tends to 1 when the PE of highest charge density between the two partners is in default (as for the PLL/PAMAMPS 100% system at saturation of the PAMAMPS substrate, PAMAMPS 100% being more densely charged than PLL); while the *n*_(Lys/AMPS)_ stoichiometry increases when the PE of highest charge density is in excess (as for the PLL/PAMAMPS 15% system at saturation of the PAMAMPS substrate, PLL being more densely charged than PAMAMPS 15%). 

### 4.2. Influence of the Ionic Strength on the Binding Constants

**Figure 4:**
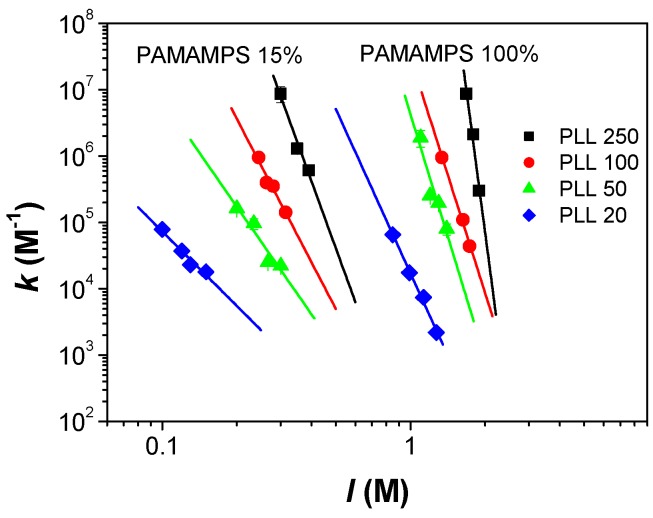
Variation of the binding site constant *k* as a function of the ionic strength for the interactions between PAMAMPS 100% or PAMAMPS 15% with PLL of different degree of polymerization (20, 50, 100, 250).

Binding site constants *k* between a PLL chain and an interacting site on a PAMAMPS chain (*–s*) were measured for different degrees of polymerization of PLL and at different ionic strengths. As illustrated in Figure 4, the binding site constants decreased linearly with the ionic strength according to a double logarithmic law. This linear double logarithmic dependence was first introduced by Lohman et al. [48,51,54] for the interactions between DNA and oligopeptides, and was observed in many polyelectrolyte-protein interactions studies [38,55,56,57], including PAMAMPS/PLL system [33,47]. This linear log-log dependence reflects the entropic contribution of the association, which is considered as the main driving force of the PEC formation. 

For a given ionic strength and whatever the PAMAMPS chemical charge density, the binding site constants *k* were found to increase with the PLL molar mass. This is due to the increase of the size *l* of the binding site *–s* with the degree of polymerization *DP^+^* of the PLL. *l* can be calculated according to [33]:

(10) $$l=b\times \frac{DP^{+}}{f\times n_{\left(\mathrm{Lys}/\mathrm{AMPS}\right)}}$$

where *b* is the length of a vinylic monomer (*b* = 0.25 nm) and *n*_(Lys/AMPS)_ is the stoichiometry expressed in charge ratio. *l* varies from 5 nm (for *DP^+^* = 20) up to 63 nm (for *DP^+^* = 250) for PAMAMPS 100%; and from 17 nm (for *DP*^+^ = 20) up to 210 nm (for *DP*^+^ = 250) for PAMAMPS 15%. The increase of the size of the binding site means that the number of electrostatic interaction points per ligand obviously increases with the *DP^+^* of PLL. 

**Figure 5:**
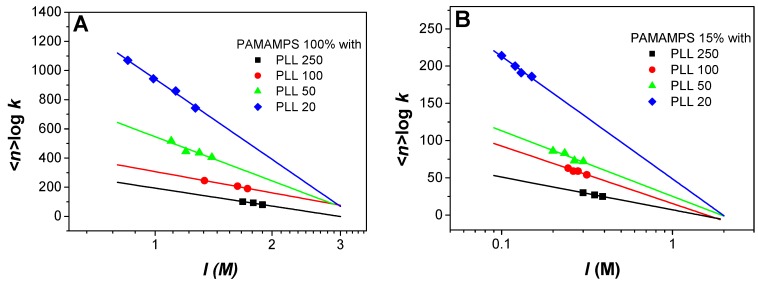
Variation of the logarithm of global constant (log β*_n_*) as a function of the logarithm of the ionic strength *I* for the interactions between PAMAMPS 100% (**A**) or PAMAMPS 15% (**B**) and PLL with different degrees of polymerization (20, 50, 100, 250).

The logarithm of the global binding constant relative to the formation of *SL_n_* complex was plotted versus the logarithm of the ionic strength in Figure 5. Note that, in order to limit the uncertainty on the stoichiometry, the logarithm of the global binding constants were calculated considering the average binding stoichiometries <*n*> over the different investigated ionic strengths (i.e., log β*_n_* = <*n>* × log *k*). 

**Figure 6:**
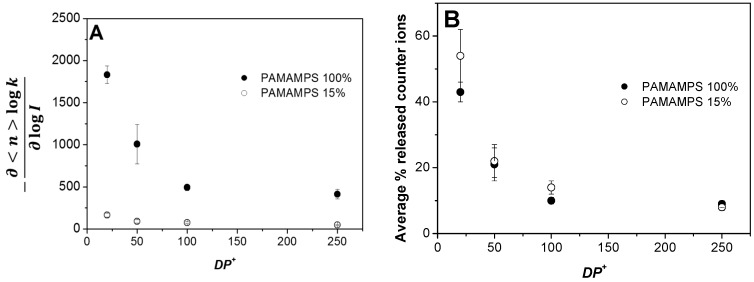
Variation of the number of released counter-ions $-\frac{\partial <n>\log k}{\partial \log I}$ (**A**) and the corresponding percentage of released counter-ions compared to the total number of condensed counter-ions (**B**) as a function of the degree of polymerization of the PLL partner (*DP^+^*) in interaction with PAMAMPS 15% or 100%. See Table 1 and Table 2 for the calculations.

The slope of the linear correlation of this log-log representation $-\frac{\partial <n>\log k}{\partial \log I}$ is a direct estimation of the number of counter-ions that are effectively released from the association of *n* PLL chains onto one PAMAMPS chain. These numerical values are reported in Table 1 and Table 2 for PAMAMPS 100% and 15%, respectively. The number of released counter-ions tends to increase for lower *DP^+^* from 410 (resp. 45) at *DP^+^* 250, up to 1830 (resp. 164) at *DP^+^* 20, for PAMAMPS 100% (resp. PAMAMPS 15%), as seen in Figure 6A. This can be explained by the creation of long PLL loops in the PEC for the higher *DP^+^*. These loops reduce the number of counter-ions that are effectively released (either from the PLL or the PAMAMPS chain). This effect was observed for both PAMAMPS 100% and PAMAMPS 15%. 

**Figure 7:**
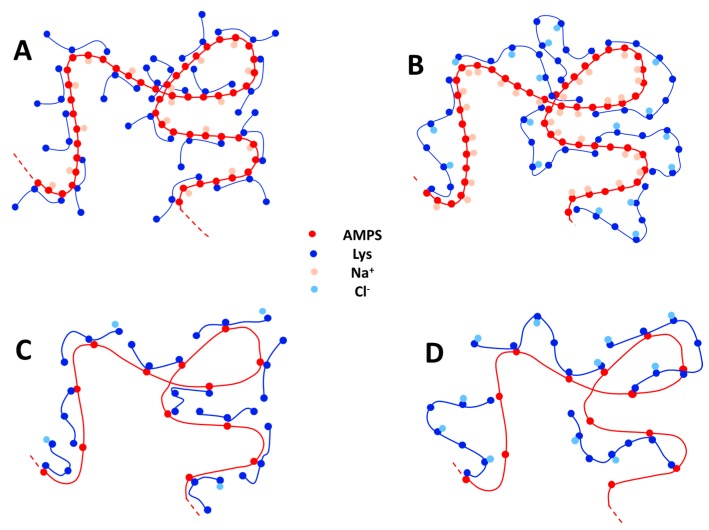
Schematic representation of a portion of PEC obtained from the association between PAMAMPS 100% with PLL20 (**A**) or with PLL 250 (**B**); and between PAMAMPS 15% with PLL20 (**C**) or with PLL 250 (**D**). Only the charged monomers and the condensed counter-ions are represented. For convenience in the representation, the PLL chain length on the picture is much lower than in reality.

To better describe this situation, a schematic representation of the formed PEC is represented in Figure 7, where only the condensed counter-ions and the monomers are presented. Short PLL chains (Figure 7A,C) allows a better overlapping between the oppositely charged chains, thus increasing the quantity of released counter-ions. For long PLL chains (Figure 7B,D), the PEC are rich in condensed counter-ions and the charge stoichiometry is reached with a statistical charge compensation that is far from the picture of ion pairs in a “ladder structure” between the oppositely charged PE. The number of released counter-ions is much higher for PAMAMPS 100% than for PAMAMPS 15%. This is due to higher initial number of condensed counter-ions (*N*_counter-ions_) before the PEC formation, which is typically about 4300–4800 for PAMAMPS 100%/PLL system and only 300–560 for PAMAMPS 15%/PLL system, as estimated from the Manning theory using Equation (9). Interestingly, the fraction of released counter-ions, which can be calculated from the $\left(-\frac{\partial <n>\log k}{\partial \log I}/N_{\mathrm{counter}\;\mathrm{ion}}\right)$ ratio, are similar (8% vs. 9% for PLL 250; 14% vs. 10% for PLL100; 22% vs. 21% for PLL 50; 54% vs. 43% for PLL 20) for both PAMAMPS charge densities for a given *DP^+^* (see Figure 6B and Table 1 and Table 2). As can be seen in Figure 6B, the fraction of released counter-ions decreases significantly with increasing *DP^+^* and seems to reach a limiting value about 8%–9% for long PLL chains. These findings could be used to estimate the fraction of released counter-ions for PLL of any *DP^+^* in interaction with a PAMAMPS of any chemical charge density. 

## 5. Conclusions

The effect of the PLL molar mass on the binding site constant and stoichiometry of PLL-PAMAMPS complexes was systematically investigated by FACCE for different ionic strengths. Increasing the molar mass of the PLL chain had two quite intuitive effects: (i) the binding site constants increased due to an increase of the number of electrostatic anchoring points; and (ii) the stoichiometry of interaction *n* decreased. More subtle effects were observed regarding the ionic strength dependence of the binding constant. This dependence, which is directly related to the total number of released counter-ions after the formation of a (1:*n*) PEC, was found to decrease with increasing PLL molar mass. It was however strongly dependent on the PAMAMPS charge density: higher charge density leading to higher number of released counter-ions. Interestingly, the fraction of released counter-ion compared to the initial total number of condensed counter-ions was almost independent of the PAMAMPS charge density and decreased from 43%–54% for *DP^+^* 20 down to 8%–9% for *DP^+^* 250. We believe that these findings could be useful for the prediction of the binding constant according to the degree of polymerization and charge density of the polyelectrolyte partners.

## Figures and Tables

**Table 1:** Physico-chemical properties of oppositely charged polyelectrolytes (PLL and PAMAMPS 100%) and the corresponding parameters of the interactions obtained by FACCE for different *DP^+^* of the PLL partner. All these parameters were determined according to Equations (7)–(9) or by curve fitting of the isotherms of adsorption (see Figures S1–S4 in the Supplementary Materials).

*f* (%)	θ^− (a)^	*DP_AMPS_* ^(b)^	*N*_Na_^+ (c)^	*DP^+^*	θ^+ (d)^	*N*_Cl_^− (e)^	*I* (M)	*n*	<*n*>	*N* (Lys/AMPS)	*N*_counter-ions_^(f)^	$-\frac{\partial \langle n\rangle logk}{\partial logI}$	Average % of released counter-ions
100	0.65	4170	2711	250	0.50	125	1.68	14	15	0.86	4503	412 ± 57	9 ± 1
							1.79	14		0.84	4459		
							1.89	15		0.91	4614		
				100	0.50	50	1.34	42	44	0.98	4805	492 ± 37	10 ± 1
							1.63	41		0.97	4780		
							1.73	48		1.13	5130		
				50	0.50	25	1.1	80	82	1.01	4718	1008 ± 233	21 ± 5
							1.2	81		0.97	4737		
							1.3	88		1.05	4904		
							1.4	80		0.96	4715		
				20	0.35	7	0.85	220	222	1.06	4251	1831 ± 104	43 ± 3
							0.99	216		1.04	4224		
							1.13	235		1.17	4359		
							1.27	218		1.00	4234		

^(a)^ see reference [43]; ^(b)^ see reference [52]; ^(c)^ calculated according to Equation (7); ^(d)^ see reference [42]; ^(e)^ calculated according to Equation (8); ^(f)^ calculated according to Equation (9).

**Table 2:** Physico-chemical properties of oppositely charged polyelectrolytes (PLL and PAMAMPS 15%) and the corresponding parameters of the interactions obtained by FACCE for different *DP^+^* of PLL partner. All these parameters were determined according to Equations (7)–(9) or by curve fitting of the isotherms of adsorption (see Figures S5–S8 in the Supplementary Materials).

*f* (%)	θ^− (a)^	*DP_AMPS_* ^(b)^	*N*_Na_^+ (c)^	*DP^+^*	θ^+ (d)^	*N*_Cl_^− (e)^	*I* (M)	*n*	<*n*>	*n* (Lys/AMPS)	*N*_counter-ions_^(f)^	$-\frac{\partial \langle n\rangle logk}{\partial logI}$	Average % of released counter-ions
15	0	510	0	250	0.50	125	0.3	4	4	2.0	512	45 ± 6	8 ± 1
							0.35	5		2.2	563		
							0.39	4		2.2	554		
				100	0.50	50	0.245	11	11	2.0	530	76 ± 12	14 ± 2
							0.263	11		2.0	531		
							0.28	11		2.1	540		
							0.315	10		1.9	510		
				50	0.50	25	0.2	16	17	1.6	403	90 ± 18	22 ± 5
							0.234	19		1.9	476		
							0.267	16		1.6	397		
							0.301	15		1.5	382		
				20	0.35	7	0.1	42	44	1.6	294	164 ± 24	54 ± 8
							0.12	42		1.7	296		
							0.13	45		1.8	318		
							0.15	46		1.8	319		

^(a)^ see reference [43]; ^(b)^ see reference [52] ; ^(c)^ calculated according to Equation (7); ^(d)^ see reference [42]; ^(e)^ calculated according to Equation (8); ^(f)^ calculated according to Equation (9).

## Supplementary Materials

The following are available online at www.mdpi.com/2073-4360/9/2/50/s1. Table S1: Concentrations of oppositely charged polyelectrolytes solutions for the preparation of PLL-PAMAMPS 100% mixtures. Table S2: Concentrations of oppositely charged polyelectrolytes solutions for the preparation of PLL-PAMAMPS 15% mixtures. Figure S1: Isotherms of adsorption and non-linear curve fitting obtained for the interactions between PLL250 and PAMAMPS 100% at different ionic strengths as noticed on the graph. Experimental conditions: PDADMAC coated capillary 33.5 cm (8.5 cm to the detector) × 50 µm i.d. Background electrolyte: 12 mM Tris, 10 mM HCl and appropriate amount of NaCl, pH 7.4. Applied voltage: +1 kV with a co-hydrodynamic pressure of +5 mbar. Detection at 200 nm. Samples were prepared in the background electrolyte by 50/50 *v*/*v* dilution of PAMAMPS and PLL solutions according to Table S1. Figure S2: Isotherms of adsorption and non-linear curve fitting obtained for the interactions between PLL100 and PAMAMPS 100% at different ionic strengths as noticed on the graph. Experimental conditions were the same as in Figure S1. Figure S3: Isotherms of adsorption and non-linear curve fitting obtained for the interactions between PLL50 and PAMAMPS 100% at different ionic strengths as noticed on the graph. Experimental conditions were the same as in Figure S1. Figure S4: Isotherms of adsorption and non-linear curve fitting obtained for the interactions between PLL20 and PAMAMPS 100% at different ionic strengths as noticed on the graph. Experimental conditions were the same as in Figure S1. Figure S5: Isotherms of adsorption and non-linear curve fitting obtained for the interactions between PLL250 and PAMAMPS 15% at different ionic strengths as noticed on the graph. Experimental conditions were the same as in Figure S1. Figure S6: Isotherms of adsorption and non-linear curve fitting obtained for the interactions between PLL100 and PAMAMPS 15% at different ionic strengths as noticed on the graph. Experimental conditions were the same as in Figure S1. Figure S7: Isotherms of adsorption and non-linear curve fitting obtained for the interactions between PLL50 and PAMAMPS 15% at different ionic strengths as noticed on the graph. Experimental conditions were the same as in Figure S1. Figure S8: Isotherms of adsorption and non-linear curve fitting obtained for the interactions between PLL20 and PAMAMPS 15% at different ionic strengths as noticed on the graph. Experimental conditions were the same as in Figure S1.
