# Supplementary material for: The Effect of Molar Mass and Charge Density on the Formation of Complexes between Oppositely Charged Polyelectrolytes
Source: Polymers (Basel). 2017 Feb 4;9(2):50. doi: 10.3390/polym9020050 (PMC6432040; doi:10.3390/polym9020050)
Supplement: Supplementary file 1 [file polymers-09-00050-s001.pdf]

Supplementary Materials: The Effect of Molar Mass and Charge Density on the Formation of Complexes between Oppositely Charged Polyelectrolytes

Feriel Meriem Lounis, Joseph Chamieh, Laurent Leclercq, Philippe Gonzalez and Hervé Cottet

1. Preparation of the PEC Mixtures

The concentrations of the oppositely charged polyelectrolytes for the preparation of PLL-PAMAMPS mixtures as well as the investigated ionic strengths are summarized in Tables S1 and S2.

Table S1. Concentrations of oppositely charged polyelectrolytes solutions for the preparation of PLL-PAMAMPS 100% mixtures.

DP^{+}	I (M)	[PAMAMPS] Stock Solution (g/L) (*)	[PLL] Stock Solution (g/L) (*)	[PLL] Diluted Solutions (g/L) (*)	$V_{PAMAMPS}$ (μL) [(a)]	V_{PLL} (μL) [(b)]	V_f (μL) [(c)]
250	1.68	1.14	5	2.5; 2; 1.5; 1.25; 1; 0.8; 0.6; 0.5; 0.4; 0.3; 0.2	100	100	200
	1.79			2.5; 2; 1.5; 1.25; 1; 0.8; 0.6; 0.5; 0.4; 0.3; 0.2			
	1.89			2.5; 2; 1.5; 1.25; 1; 0.8; 0.6; 0.5; 0.4; 0.3; 0.2			
100	1.34			2.5; 2; 1.5; 1.25; 1; 0.8; 0.6; 0.5; 0.4; 0.3			
	1.63			2.5; 2; 1.5; 1.25; 1; 0.8; 0.6; 0.5; 0.4; 0.3; 0.2			
	1.73			2.5;2; 1.5;1.25; 1; 0.8; 0.6; 0.5; 0.4; 0.3; 0.2; 0.1			
50	1.1			3; 2.5; 2; 1.6; 1.2; 1; 0.8; 0.6			
	1.2			3; 2; 1.6; 1.2; 0.8; 0.7; 0.6; 0.5; 0.4			
	1.3			2; 1.6; 1.2; 0.8; 0.7; 0.5			
	1.4			4; 3; 2; 1.6; 1.2; 1; 0.8			
20	0.85			2.5; 2; 1.5; 1.25; 1; 0.8; 0.6; 0.5; 0.4; 0.3; 0.2			
	1.99			2.5; 2; 1.5;1.25; 1; 0.8; 0.6;0.5; 0.4; 0.3;0.2; 0.1			
	1.13			2.5; 2;1.5; 1.25; 1; 0.8;0.6; 0.5; 0.4;0.3; 0.2; 0.1			
	1.27			1.5; 1.25; 1: 0.8; 0.6; 0.5; 0.4; 0.3; 0.2; 0.1			

(*) For each FACCE experiment, PAMAMPS and PLL stock solutions as well as PLL diluted solutions were prepared in the same Tris-HCl-NaCl buffer pH 7.4 (12 mM Tris, 10 mM HCl and appropriate amount of NaCl to adjust the ionic strength of the medium); [(a)] Volume of PAMAMPS stock solution in the mixtures; [(b)] Volume of PLL diluted solutions in the mixtures; [(c)] final volume of the mixtures.

Table S2. Concentrations of oppositely charged polyelectrolytes solutions for the preparation of PLL-PAMAMPS 15% mixtures.

DP^{+}	*I* (M)	[PAMAMPS] Stock Solution (g/L) (*)	[PLL] Stock Solution (g/L) (*)	[PLL] Diluted Solutions (g/L) (*)	$V_{PAMAMPS}$ (μL) [(a)]	V_{PLL} (μL) [(b)]	V_f (μL) [(c)]
250	0.3	3.2	5	2.5; 2; 1.5; 1.25; 1; 0.8; 0.6	100	100	200
	0.32			2.5; 2; 1.5; 1.25; 1; 0.8; 0.6; 0.5; 0.4; 0.3			
	0.39			2.5; 2; 1.5; 1.25; 1; 0.8; 0.6; 0.5; 0.4; 0.3			
100	0.245			2.5; 2; 1.5; 1; 0.8; 0.6; 0.5; 0.4; 0.3			
	0.263			2.5; 2; 1.5; 1.25; 1: 0.8; 0.6; 0.5; 0.4; 0.3			
	0.28			2.5; 2; 1.5; 1.25; 1; 0.8; 0.6; 0.5; 0.4; 0.3; 0.2			
	0.315			2.5; 2; 1.5; 1.25; 1; 0.8; 0.6; 0.5; 0.4; 0.3; 0.2			

50	0.2	4; 3; 2.5; 2; 1.6; 1.2; 1; 0.8
	0.234	3; 2.5; 2; 1.6; 1.2; 1; 0.8; 0.6; 0.4
	0.267	3; 2.5; 2; 1.6; 1.2; 1; 0.8; 0.6; 0.4
	0.301	4; 3; 2.5; 2; 1.6; 1.2; 1; 0.8; 0.6; 0.4; 0.2
20	0.1	2.5; 2; 1.5; 1.25; 1; 0.8; 0.6; 0.5; 0.4; 0.3; 0.2
	0.12	2.5; 2; 1.5; 1.25; 1; 0.8; 0.6; 0.5; 0.4; 0.3; 0.2
	0.13	2.5; 2; 1.5; 1.25; 1; 0.8; 0.6; 0.5; 0.4; 0.3; 0.2
	0.15	2.5; 2; 1.5; 1.25; 1; 0.8; 0.6; 0.5; 0.4; 0.3; 0.2

(*) For each FACCE experiment, PAMAMPS and PLL stock solutions as well as PLL diluted solutions were prepared in the same Tris-HCl-NaCl buffer pH 7.4 (12 mM Tris, 10 mM HCl and appropriate amount of NaCl to adjust the ionic strength of the medium). [(a)] Volume of PAMAMPS stock solution in the mixtures; [(b)] Volume of PLL diluted solutions in the mixtures; [(c)] final volume of the mixtures.

2. Isotherms of Adsorption

The influence of the ionic strength and the chemical charge density on the thermodynamic parameters on the interactions between PLL and PAMAMPS was studied by plotting 29 isotherms of adsorption displayed in Figures S1–S8 with the non-linear curve fitting allowing the determination of the binding site constants and the chain stoichiometries.

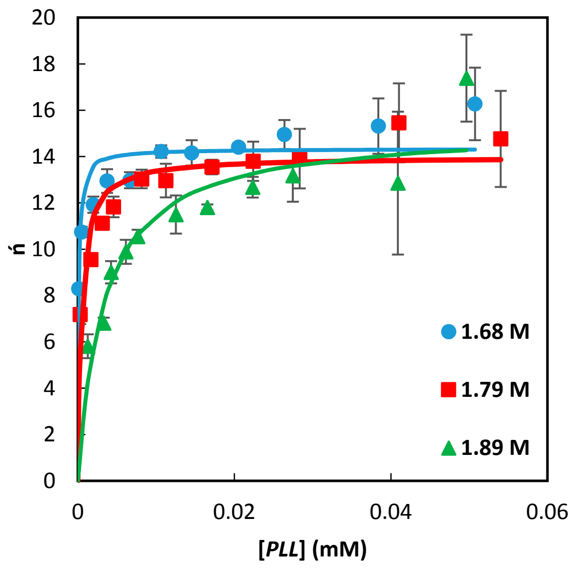

Figure S1. Isotherms of adsorption and non-linear curve fitting obtained for the interactions between PLL250 and PAMAMPS 100% at different ionic strengths as noticed on the graph. Experimental conditions: PDADMAC coated capillary 33.5 cm (8.5 cm to the detector) × 50 µm i.d. Background electrolyte: 12 mM Tris, 10 mM HCl and appropriate amount of NaCl, pH 7.4. Applied voltage: +1 kV with a co-hydrodynamic pressure of +5 mbar. Detection at 200 nm. Samples were prepared in the background electrolyte by 50/50 *v*/*v* dilution of PAMAMPS and PLL solutions according to Table S1.

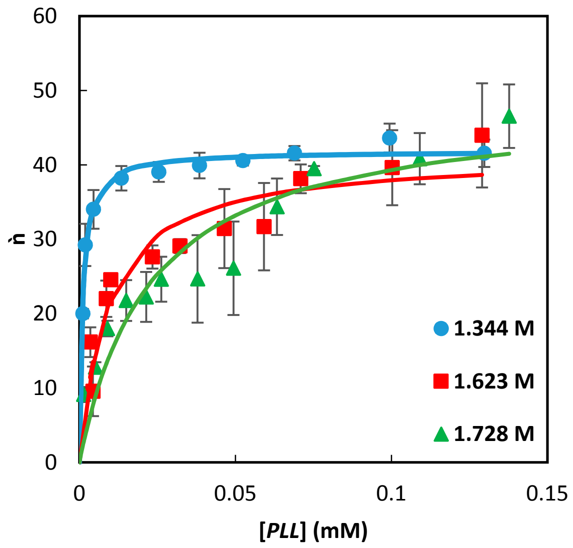

Figure S2. Isotherms of adsorption and non-linear curve fitting obtained for the interactions between PLL100 and PAMAMPS 100% at different ionic strengths as noticed on the graph. Experimental conditions were the same as in Figure S1.

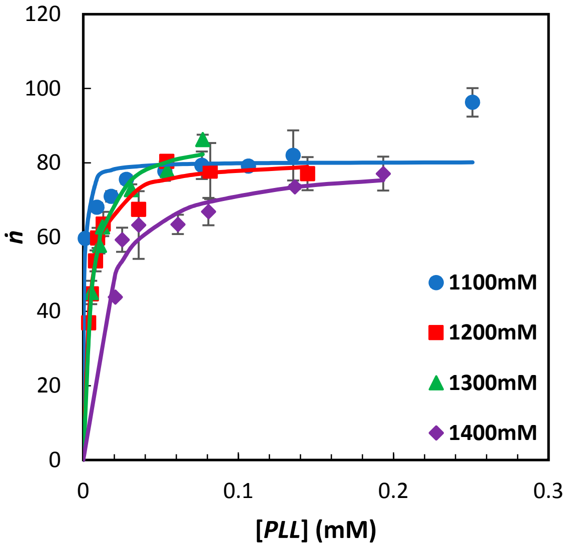

Figure S3. Isotherms of adsorption and non-linear curve fitting obtained for the interactions between PLL50 and PAMAMPS 100% at different ionic strengths as noticed on the graph. Experimental conditions were the same as in Figure S1.

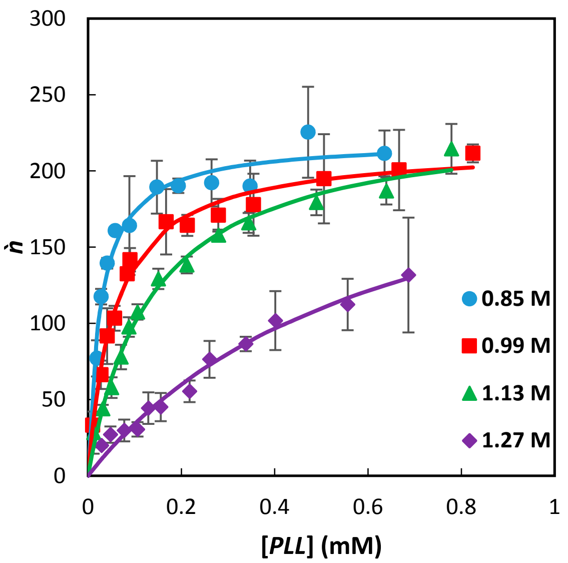

Figure S4. Isotherms of adsorption and non-linear curve fitting obtained for the interactions between PLL20 and PAMAMPS 100% at different ionic strengths as noticed on the graph. Experimental conditions were the same as in Figure S1.

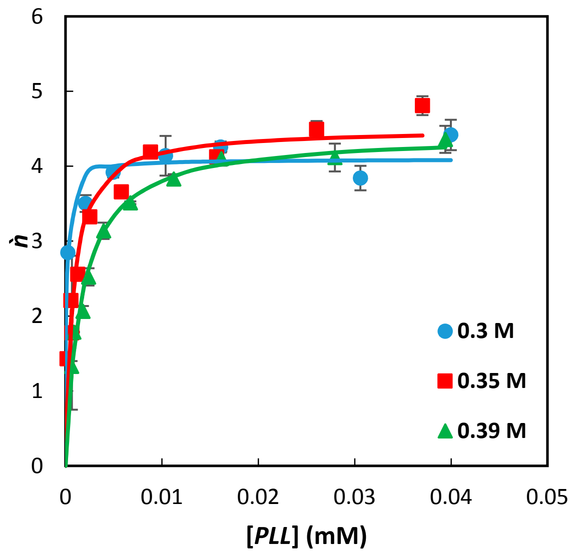

Figure S5. Isotherms of adsorption and non-linear curve fitting obtained for the interactions between PLL250 and PAMAMPS 15% at different ionic strengths as noticed on the graph. Experimental conditions were the same as in Figure S1.

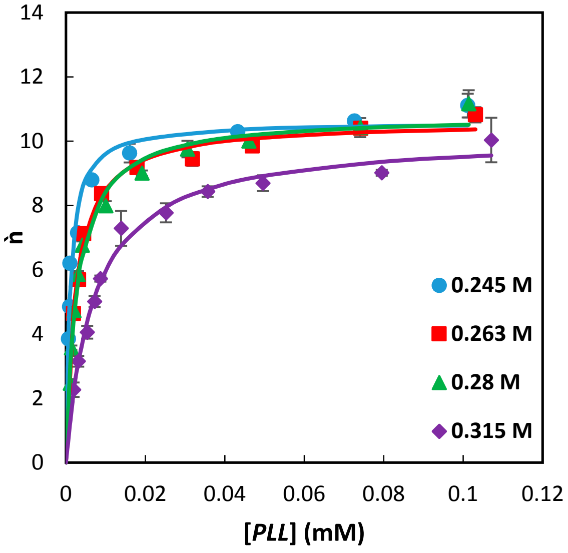

Figure S6. Isotherms of adsorption and non-linear curve fitting obtained for the interactions between PLL100 and PAMAMPS 15% at different ionic strengths as noticed on the graph. Experimental conditions were the same as in Figure S1.

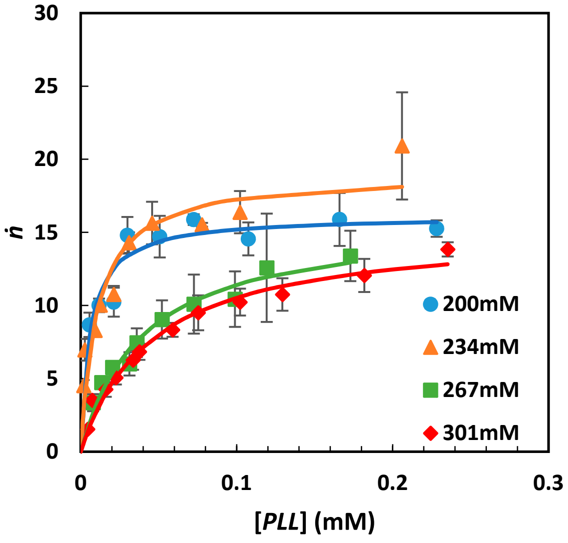

Figure S7. Isotherms of adsorption and non-linear curve fitting obtained for the interactions between PLL50 and PAMAMPS 15% at different ionic strengths as noticed on the graph. Experimental conditions were the same as in Figure S1.

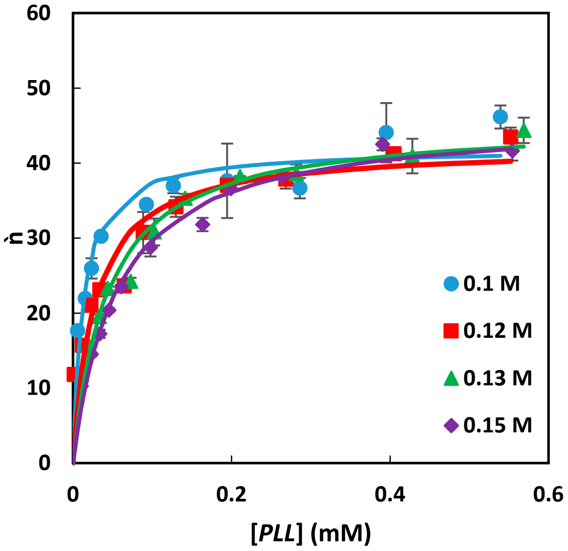

Figure S8. Isotherms of adsorption and non-linear curve fitting obtained for the interactions between PLL20 and PAMAMPS 15% at different ionic strengths as noticed on the graph. Experimental conditions were the same as in Figure S1.
